# Non-Coding RNAs as Emerging Biomarkers in Leishmaniasis and Chagas Disease

**DOI:** 10.3390/tropicalmed10110319

**Published:** 2025-11-13

**Authors:** Eduardo Ramos Juárez, Eduardo Pérez-Campos Mayoral, Laura Pérez-Campos Mayoral, Adriana Moreno Rodríguez, Carlos Romero-Díaz, Miriam Emily Avendaño-Villegas, Tania Sinaí Santiago Ramírez, Margarito Martínez Cruz, José Luis Hernández-Morales, Lilian Guadalupe Bolaños-Hilario, Iam Kevin Suárez Luna, Jesús Elizarrarás-Rivas, Aldo Abel García González, Hector Alejandro Cabrera-Fuentes, María Teresa Hernández-Huerta, Eduardo Pérez-Campos

**Affiliations:** 1 División de Estudios de Posgrado e Investigación, Tecnológico Nacional de Mexico/Instituto Tecnológico de Oaxaca, Oaxaca 68030, Mexico; ramosjuarez9785@gmail.com (E.R.J.); carlos.romero@itoaxaca.edu.mx (C.R.-D.); e_mily_3@hotmail.com (M.E.A.-V.); mcruz@itoaxaca.edu.mx (M.M.C.); liliangbh@gmail.com (L.G.B.-H.); 2 Centro de Investigación Facultad de Medicina UNAM-UABJO, Facultad de Medicina y Cirugía, Universidad Autónoma “Benito Juárez” de Oaxaca, Oaxaca 68020, Mexico; eperezcampos.fmc@uabjo.mx (E.P.-C.M.); lperezcampos.fmc@uabjo.mx (L.P.-C.M.); joseluishmorales@gmail.com (J.L.H.-M.); kevinlevin61466@gmail.com (I.K.S.L.); aldo_garciagonzalez@hotmail.com (A.A.G.G.); hafuentes@iau.edu.sa (H.A.C.-F.); 3 Laboratorio de Estudios Epidemiológicos, Clínicos, Diseños Experimentales e Investigación, Facultad de Ciencias Químicas, Universidad Autónoma “Benito Juárez” de Oaxaca, Oaxaca 68120, Mexico; arimor10@cecad-uabjo.mx; 4 Facultad de Medicina y Cirugía, Benemérita Universidad de Oaxaca, Oaxaca 68020, Mexico; ttanniaramirez9@gmail.com; 5 Coordinación de Investigación en Salud, IMSS, Oaxaca 68040, Mexico; dr.jesuselizarraras@gmail.com; 6 Hospital General de Zona No.1 Dr. Demetrio Mayoral Pardo, IMSS, Oaxaca 68040, Mexico; 7 Facultad de Medicina y Cirugía, Universidad Autónoma “Benito Juárez” de Oaxaca, Oaxaca 68020, Mexico; 8 División de Estudios de Posgrado e Investigación, Tecnológico Nacional de Mexico/Instituto Tecnológico de Tijuana, Tijuana 22414, Mexico; 9 R&D Group, Vice Presidency Scientific Research & Innovation, Imam Abdulrahman Bin Faisal University (IAU), Dammam 31441, P.O. Box. 1982, Saudi Arabia; 10 Dirección de la División de Investigación y Desarrollo Científico, Benemérita Universidad de Oaxaca, Oaxaca 68000, Mexico; 11 SECIHTI, Facultad de Medicina y Cirugía, Universidad Autónoma “Benito Juárez” de Oaxaca, Oaxaca 68020, Mexico

**Keywords:** leishmaniasis, chagas disease, non-coding RNAs, biomarkers, extracellular vesicles

## Abstract

Leishmaniasis and Chagas disease, caused by *Leishmania* spp. and *Trypanosoma cruzi*, are neglected tropical diseases with significant global health burden, particularly in resource-limited regions. Despite their impact, diagnosis and treatment remain challenging due to limited diagnostic tools and the toxicity of available therapies. Our objective is to propose the incorporation of markers for the diagnosis of leishmaniasis and Chagas disease using ncRNA. This narrative review evaluates studies published between 2010 and 2024 (PubMed, Scopus, Google Scholar) using the SANRA scale to assess the potential of non-coding RNAs (ncRNAs) as biomarkers for these infections. Both parasites release small RNAs via extracellular vesicles that modulate host–pathogen interactions and gene expression. Although RNA interference machinery is absent in *T. cruzi* and most *Leishmania* species, it persists in early-diverging lineages. In leishmaniasis, distinct miRNA expression profiles—including miR-155-5p, miR-5011-5p, miR-6785-5p, and miR-361-3p—demonstrate high diagnostic accuracy for detecting infection (AUC up to 1.0). Serum long ncRNAs such as MALAT1 and NUTM2A-AS1 show potential diagnostic value, though clinical validation remains pending. For Chagas disease, the available evidence on ncRNAs primarily addresses the diagnosis of clinical manifestations rather than initial infection. Host miRNAs, including miR-21, miR-145, miR-146a/b, and miR-19a-3p, correlate with cardiac involvement, immune dysregulation, and inflammation during chronic *T. cruzi* infection. Circulating miRNAs exhibit modest sensitivity (57–67%) and specificity (57–80%) for diagnosing chronic Chagas cardiomyopathy, indicating their utility in assessing disease progression and organ damage rather than detecting early infection. This review distinguishes between ncRNAs that diagnose infection and those that evaluate disease severity or organ involvement. Altered ncRNA expression profiles represent promising biomarkers for species differentiation, treatment monitoring, and assessing cardiac complications in Chagas disease, with broader diagnostic applications emerging for leishmaniasis.

## 1. Introduction

Leishmaniasis and American trypanosomiasis are diseases caused by protozoan parasites and are considered neglected tropical diseases. The etiological agent of leishmaniasis is a different species of the genus Leishmania, whereas for Trypanosomiasis, also known as Chagas disease, it is the parasite *Trypanosoma cruzi* (*T. cruzi*). According to the World Health Organization (WHO), between 700,000 and 1 million new cases of leishmaniasis are reported annually, with an estimated 12 million infected individuals worldwide. In comparison, Chagas disease affects between 6 and 8 million people, causing 12,000 deaths per year [1].

In Mexico, leishmaniasis, also known as “gum ulcer,” was first recorded in 1912 among individuals who collected chewing gum from the Manilkara zapota tree. The first cases of Chagas disease were reported in 1940 in two patients from Oaxaca [2].

Globally, leishmaniasis and Chagas disease mainly affect developing countries, with a greater impact in rural areas and tropical and subtropical regions. In Africa, Asia, and Latin America, cutaneous and visceral leishmaniasis continues to be a significant public health problem, with many cases undiagnosed or poorly treated. For example, in the Amazon region, Brazil is one of the countries with the highest burden of visceral leishmaniasis, while India, Pakistan, and Afghanistan have high rates of cutaneous leishmaniasis [1].

Chagas disease remains endemic in Latin America, particularly in Brazil, Bolivia, Argentina, and Mexico, where prevalence is highest. Migration patterns have introduced the disease to non-endemic regions, including the United States, Europe, Africa, and Asia. This geographic expansion has transformed Chagas disease from a regional problem into a global health threat, requiring international surveillance and control strategies [3].

The transmission of these diseases can occur through the bite of vector insects (such as bed bugs and sandflies), via blood transfusion, or organ transplant. In the case of Trypanosomiasis, it can also be spread by ingesting food contaminated with parasites. Among the strategies for reducing cases are prevention strategies, which include informing the population about the disease and its vectors, as well as screening blood banks to prevent the use of contaminated blood [4].

Leishmaniasis manifests with diverse clinical symptoms depending on the causative parasite. Cutaneous leishmaniasis presents skin lesions, including ulcers, smooth nodules, and wart-like lesions. In contrast, visceral leishmaniasis primarily presents symptoms such as fever, weight loss, anemia, abdominal distension, splenomegaly, and hepatomegaly [3]. Chagas disease progresses through acute and chronic phases. The acute phase can manifest as chagoma of inoculation, fever, lymphadenopathy, anemia, musculoskeletal pain, heart failure, and meningoencephalitis. The chronic phase typically involves cardiac abnormalities, including heart failure, arrhythmias, and ventricular aneurysms, alongside gastrointestinal issues like megaesophagus and megacolon [4].

For the diagnosis of leishmaniasis, biopsies and cultures are performed on the spleen, bone marrow, liver, lymph nodes, and skin; the latter is a widely used option in cases of cutaneous leishmaniasis. In addition, serological tests such as indirect agglutination and indirect immunofluorescent antibody tests, as well as polymerase chain reaction (PCR) tests for Leishmania, and direct tests for detecting the parasite in blood by microscopy (the fat drop test), are also used [5].

While parasitological methods in blood can detect *Trypanosoma cruzi* in the acute phase, in the chronic phase, the diagnosis depends on clinical evaluation, serological tests, such as enzyme-linked immunosorbent (ELISA) assays, indirect immunofluorescence, or indirect hemagglutination, and epidemiological history [6,7,8]. In recent years, nucleic acid amplification techniques, such as real-time PCR, have been implemented in some countries, particularly for the early diagnosis of congenital Chagas disease in infants, as well as for treatment monitoring in the context of clinical trials [9,10].

Detection methods for these diseases do not define the progression of infection in patients; however, the use of biomarkers can serve as a more accurate detection method, offering an advantage in the treatment of these diseases by detecting infections during their early stages and determining the status of the infection.

Non-coding RNAs (ncRNAs) have emerged as potential biomarkers for leishmaniasis and Chagas disease, although the available evidence primarily addresses different diagnostic applications. For leishmaniasis, ncRNAs show promise in detecting active infection, while for Chagas disease, current research focuses mainly on identifying cardiac involvement during the chronic phase rather than initial infection. This review evaluates the diagnostic potential of ncRNAs—including microRNAs (miRNAs), long non-coding RNAs (lncRNAs), PIWI-interacting RNAs (piRNAs), short interfering RNAs (siRNAs), and tRNA-derived small RNAs (tRFs)—as biomarkers for infections caused by Leishmania spp. and *T. cruzi*. We distinguish between ncRNAs that detect infection and those that assess disease progression or organ involvement, particularly cardiac manifestations in chronic Chagas disease. A comprehensive literature search was conducted across PubMed, Scopus, and Google Scholar databases. The search strategy incorporated a combination of keywords and Medical Subject Headings (MeSH) terms, including “Leishmania,” “Leishmaniasis,” “Trypanosoma cruzi,” “Chagas disease,” “ncRNA,” “non-coding RNA,” “lncRNA,” “miRNA,” “piRNA” and “siRNAs”. Inclusion criteria focused on original research and review articles relevant to the stated objective. The quality of included articles was independently assessed using the SANRA (Scale for the Assessment of Narrative Review Articles) quality assessment tool.

## 2. Clinical Diagnosis of Leishmaniasis and Trypanosomiasis

Multiple laboratory tests are available to diagnose leishmaniasis and trypanosomiasis, as accurate detection often requires a combination of techniques to maximize the probability of a positive result—such as microscopy, molecular methods, and serology—to enhance sensitivity, specificity, and species identification.

The three primary clinical syndromes caused by infection with *Leishmania* parasites are cutaneous, mucocutaneous, and visceral (also known as kala-azar) leishmaniasis. The diagnosis of visceral leishmaniasis (VL) relies on a combination of clinical signs and parasitological or serological tests. Serological tests are of limited utility in cases of cutaneous and mucocutaneous leishmaniasis, where the diagnosis is confirmed when parasitological analyses support the clinical manifestations [1].

Moreover, many of the regional recommendations issued by the WHO and Pan American Health Organization (PAHO) are based on observational studies, case series, and expert reports. Therefore, the diagnostic approach to leishmaniasis should be based on the combined application of different laboratory tests. Generally, various methods are used for diagnosing leishmaniasis, including the visualization of characteristic amastigotes in smears or tissue samples, the isolation of the parasite through in vitro culture, and the molecular detection of parasitic DNA. Additionally, for individuals with suspected visceral leishmaniasis, serological tests are used when direct diagnostic techniques (such as microscopy, culture, or molecular tests) cannot be performed or when these yield negative results. However, anti-leishmanial antibodies may persist for years after clinically successful therapy or, conversely, may be undetectable or present at low levels in patients with VL who are immunosuppressed due to Human Immunodeficiency Virus/Acquired Immunodeficiency Syndrome (HIV/AIDS) or other causes. This variability, combined with the potential for false-negative results, limits the diagnostic utility of serological assays in this context, Table 1 [11].

The diagnosis of acute Chagas infections is performed through the identification of trypomastigotes in the blood using direct microscopy. This involves preparing and staining thick and thin blood smears to visualize the parasites. However, blood parasite levels decrease rapidly within a few months, becoming undetectable with parasitological methods during the chronic phase of the disease. For the diagnosis of Chagas disease in the chronic phase, the detection of antibodies against the parasite *T. cruzi* is required using serological tests. No single test is sufficiently sensitive or specific to confirm the diagnosis; therefore, it is recommended to use two or more serological tests that detect antibodies against different antigens. Standard techniques include the indirect hemagglutination, ELISA, and the indirect immunofluorescence antibody (IFA) test. Additionally, assessing the patient’s clinical and epidemiological history helps identify potential risk factors for infection, Table 2 [14].

## 3. Characteristics of miRNA, piRNA, and lncRNA

The genome contains both coding RNA (cRNA) and ncRNA. NcRNA is broadly categorized into long non-coding RNA (lncRNA) and small non-coding RNA (sncRNA). The main types of sncRNA include microRNA (miRNA), small interfering RNA (siRNA), PIWI-interacting RNA (piRNA), small nucleolar RNA (snoRNA), small nuclear RNA (snRNA), and tRNA-derived fragments (tRFs) [17,18]. Genome-derived microRNAs (miRNAs) and exogenous small interfering RNAs (siRNAs), the two main types of RNA interference (RNAi) molecules, both integrate into RNA-induced silencing complexes to regulate gene expression after transcription [19].

Multiple types of sncRNA regulate gene expression [20]. There are both differences and similarities between siRNA, miRNA, piRNA, and lncRNA. For example, in their nucleotide length, ssiRNAs are 20 to 25 nts, miRNAs are 21 to 23 nts, piRNAs are 26 to 32 nts, and lncRNAs are >200 nts (Table 1).

The existence of siRNAs was first reported in *Caenorhabditis elegans* in 1998 by Fire et al. [21]. These siRNAs are derived from double-stranded RNA (dsRNA) through a processing step involving Dicer/DCL enzymes [22]. Once processed, siRNAs play a critical role in guiding Argonaute proteins to mediate the precise cleavage of target RNA molecules. In human cells, siRNAs play a crucial role in gene regulation and cellular defense mechanisms. Furthermore, endogenous siRNAs have been identified in *Leishmania* species that possess an active RNAi pathway [23]. Despite this, the natural presence of an endogenous RNAi pathway in *T. cruzi* has not yet been established [24].

miRNAs were first reported in *C. elegans* by Lee et al. in 1993 [25] and were subsequently identified as small RNAs [26]. Although they constitute only 2–3% of the human genome, miRNAs are estimated to regulate the expression of over 60% of genes [27]. Their biogenesis begins with precursor transcripts processed by the RNase III enzymes Drosha and Dicer in animals, or by Dicel-like (DCL) enzymes in plants [28]. In animals, these precursors are transported from the nucleus by Exportin-5 (Exp5) to be loaded into an Argonaute (AGO) protein complex [29,30]. siRNAs and miRNAs are short-duplex molecules that have silencing functions [31].

miRNAs regulate gene expression at the post-transcriptional level through interactions with specific regions of the target RNA, utilizing the RISC complex machinery, which induces RNA destabilization and degradation [32].

Most mature primary piRNAs have a uridine at the 5’ end and a 2’-OH methyl group at the 3’ end [33]. They are transcribed from piRNA gene clusters, most of which are unidirectional in somatic cells and double-stranded in the germline [34]. Mature piRNAs associate with proteins of the PIWI-like family (MILI, MIWI, and MIWI2) to form the piRNA-induced silencing complex (piRISC) for transposon silencing [35]. In humans, these complexes induce gene silencing through the action of the PIWIL1, PIWIL2, PIWIL3, and PIWIL4 proteins. These RNAs are stable and do not degrade in circulation [36].

LncRNA have been defined as transcripts longer than 200 nucleotides that do not encode proteins [37]. LncRNAs are crucial regulators of gene expression, operating at multiple levels. They can guide chromatin-modifying complexes to specific DNA locations, allowing for epigenetic control of gene activity. Furthermore, they influence gene expression transcriptionally and post-transcriptionally by modulating processes like alternative splicing, mRNA stability, and translation [38]. Their functions extend to fundamental cellular activities, including cell differentiation, cell cycle control, and apoptosis [39].

The expression of lncRNAs is often tissue- and cell-type-specific [40]. Consequently, their dysregulation is implicated in a wide range of human diseases, including various cancers and neurodegenerative disorders, highlighting their potential as both biomarkers and therapeutic targets. While their nucleotide sequences tend to be less conserved across species than protein-coding genes, their structural elements may be evolutionarily preserved [38].

## 4. ncRNA in Kinetoplastids

**Figure 1:**
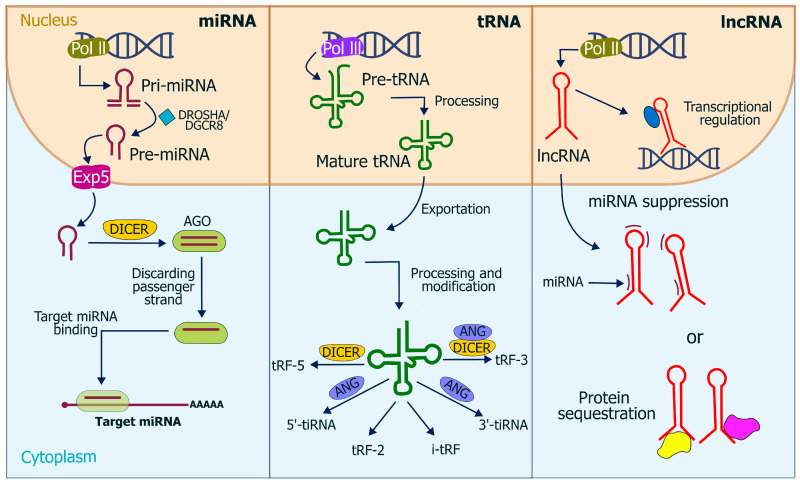
Biogenesis and functions of ncRNAs, including microRNAs (miRNAs), transfer RNAs (tRNAs), and long non-coding RNAs (lncRNAs). In the nucleus, miRNAs are transcribed by RNA polymerase II (Pol II) as primary transcripts (pri-miRNAs) and processed by the DROSHA/DGCR8 complex into precursor miRNAs (pre-miRNAs), which are exported to the cytoplasm via Exportin-5 (Exp5) and further processed by DICER to form Argonaute (AGO) complexes that regulate target mRNAs. tRNAs, transcribed by RNA polymerase III (Pol III), undergo processing and modification to produce mature tRNAs and tRNA-derived fragments (tRFs and tiRNAs) through DICER or ANG activity. lncRNAs, also transcribed by Pol II, participate in transcriptional regulation, miRNA suppression, and protein sequestration. The diagram highlights the nuclear and cytoplasmic localization of these processes.

On the other hand, *T. cruzi* utilizes ncRNAs to regulate gene expression, releasing various small RNAs through extracellular vesicles (EVs) [39,40]. This regulatory system is critical in many trypanosomatids due to the lack of a functional RNA interference (RNAi) pathway [40]. Genomic analysis confirmed the absence of critical RNAi genes, such as Dicer-like (DCL) enzymes and Argonaute (AGO), in *T. cruzi* and “higher” *Leishmania* species (subgenus *Leishmania*). This explained the lack of RNAi activity in *T. cruzi* [41]. The composition and abundance of these small RNAs differ significantly between the parasite’s life stages, suggesting diverse biological functions [39]. Among the different types of Small RNAs are the Ribosomal RNA-derived fragments (rRNA-derived, small RNAs or sdrRNAs). These are the most abundant small RNAs found in *T. cruzi* EVs, constituting 54–74% of the small RNA content in vesicles from epimastigotes (eVes) and metacyclic trypomastigotes (mVes) [42]. In whole-parasite extracts, they represent about 17% of reads in epimastigotes and 25% in metacyclic forms. Under nutritional stress, sdrRNAs become the most prevalent small RNA class in both EVs (46%) and intracellularly (58%). EVs carrying these sdrRNAs can modulate host cell gene expression and invasion [39]. The transfer RNA-derived fragments (tRNA-derived small RNAs or tsRNAs or tRFs), is the second most common class of small RNA, their abundance varies greatly by stage, making up 26–34% in epimastigotes and their vesicles, but increasing to 63% in metacyclic forms, while being lower in metacyclic-derived vesicles (6%) [42]. In epimastigotes, 89% of tsRNAs are 3’ halves with an average length of 38 nucleotides, and 75% have a CCA extension. The dominance of 5’ or 3’ derived fragments changes depending on the parasite’s life stage [39]. The small nucleolar RNA-derived fragments (snoRNA-derived small RNAs or sdRNAs) are primarily observed in metacyclic trypomastigote parental cells (4%) [42]. They have a length of approximately 35 nucleotides [39]. Other small RNAs, originating from coding sequences (CDS), are more prevalent in metacyclic-derived samples (5%) than in epimastigotes (1%). Additionally, small nuclear RNA-derived fragments (snsRNAs) have been identified, mainly from U4 and U5 snRNAs, with a median length of 40 nucleotides. Other researchers at *T. cruzi* found approximately 95% to 98% ncRNAs. These vary by haplotype and include small RNAs originating from tRNAs, rRNAs, snRNAs, and snoRNAs, Figure 1 [43].

*Leishmania* species, including *L. donovani* and *L. braziliensis*, utilize ncRNAs for gene expression regulation, with exosomal transfer RNA-derived small RNAs (tsRNAs) being frequently observed, primarily from the 5’ arm of tRNA-Asp and tRNA-Gln. Exosomes also contain small nuclear RNA (snRNA)-derived fragments and a small proportion (approximately 1.5%) of small DNA-derived RNAs (sdRNAs). Small ribosomal RNA (srRNA)-derived fragments constitute a significant portion of exosomal in *L. donovani* (31%) and *L. braziliensis* (15%), exhibiting distinct peak size distributions [40]. Unlike *Trypanosoma cruzi*, *Leishmania* did not exhibit site-specific enrichment for rRNA-derived fragments, with 90% of the mapped fragments mapping to both the 28S and 18S rRNA genes. While some *Leishmania* subgenera have lost or possess degenerated RNA interference (RNAi) genes, functional RNAi pathways involving AGO and Dicer orthologs are present in early diverging *Leishmania* subgenera like *L. braziliensis* and *L. guyanensis* [41]. In these species, 20–25 nucleotide siRNAs, mainly derived from SLACS and TATE elements, are found in exosomes, suggesting their role in intercellular communication. Experimental confirmation in *L. braziliensis* revealed small RNAs of the expected sizes that immunoprecipitated with AGO1, indicating a functional involvement similar to that of siRNAs in *T. brucei*. However, *L. braziliensis* lacks HEN1, a methyltransferase found in *T. brucei* that modifies the 3’ end of siRNAs, resulting in significant 3’ heterogeneity in *L. braziliensis* siRNAs and suggesting mechanistic diversification of the RNAi pathway within trypanosomatids.

On the other hand, in eukaryotes, non-coding RNAs, encompassing small molecules such as miRNA and piRNA, as well as lncRNA, are now recognized as master regulators in the biology of parasitic infections. They play a pivotal role in controlling parasite development, mediating host–pathogen interactions, and facilitating immune evasion, making them valuable as potential biomarkers and therapeutic targets, for example, in kinetoplastids.

*Leishmania* and *Trypanosoma cruzi*, lncRNA are key regulators in parasite development, host interaction, and immune modulation [40,44]. In addition, although specific trypanosomatid-specific PIWI-like proteins, such as TcPIWI-tryp, are known to interact with tsRNAs, their roles diverge from the canonical PIWI/Argonaute interactions seen in classical eukaryotic small RNA pathways [43,45].

Also, siRNA currently has no diagnostic application for Chagas disease or leishmaniasis. It is used in experimental settings to study gene function or in therapeutic studies. There is no evidence of its clinical or experimental use as a direct diagnostic marker for Chagas disease or leishmaniasis, and it was used here only for comparison with other ncRNAs in Table 3.

## 5. ncRNA as Diagnostic Biomarkers in Leishmaniasis

In parasitic infections, the small non-coding RNAs (sncRNAs) can act as “molecular parasites” that influence host–pathogen interactions [49]. A recent comprehensive study has significantly advanced our understanding of leishmaniasis by characterizing ncRNAs across 26 strains from 16 *Leishmania* spp. [50]. This analysis revealed conserved ncRNAs shared among different *Leishmania* spp., which proved valuable in differentiating between subgenera and species associated with visceral leishmaniasis, which will be used for diagnosis or therapeutic interventions.

Infections by *Leishmania* parasites trigger distinct changes in host miRNA expression, which are being explored for their diagnostic potential [51]. Specific miRNAs, such as miR-155 and miR-146a, are capable of distinguishing between the clinical forms of the disease [52]. Masoudsinaki et al. [53] investigated the differential expression of four specific miRNAs (miR-4795-3p, miR-6785-5p, miR-5011-5p, and miR-155-5p) in skin lesions from cutaneous leishmaniasis (CL) patients infected with *L. major* and *L. tropica*. They found a significant upregulation of miR-155-5p, miR-5011-5p, and miR-6785-5p in *L. tropica*-infected patients when compared to healthy controls. Similar, but more pronounced, expression patterns for miR-155-5p and miR-6785-5p were observed in *L. major*-infected patients, although miR-4795-3p was notably downregulated in this group. Receiver operating characteristic (ROC) analysis revealed that individual miRNAs exhibited high diagnostic potential; for example, miR-155-5p, miR-5011-5p, and miR-6785-5p effectively distinguished L. tropica-infected patients from healthy controls with high accuracy, achieving an Area Under the Curve (AUC) value of up to 1.00 (Table 4). Furthermore, combining these miRNAs further improved diagnostic efficacy, achieving high specificity (over 90%) in differentiating CL patients from healthy individuals and in distinguishing between *L. tropica* and *L. major* infections. These findings suggest that distinct miRNA expression profiles in skin lesions could serve as valuable non-invasive diagnostic tools for acute CL. Additionally, significantly elevated levels of miR-361-3p and miR-140-3p have been found in lesions compared to normal skin in CL [52]. Notably, miR-361-3p demonstrated a positive correlation with both therapeutic failure and extended healing times. Further analysis revealed the strong diagnostic potential of miR-361-3p, which accurately identified patients likely to fail initial pentavalent antimonial treatment with high sensitivity and specificity. The authors propose that the observed upregulation of miR-361-3p and miR-140-3p, which target pro-inflammatory genes like TNF and GZMB, may either represent an attempt to mitigate excessive inflammation or, conversely, contribute to tissue damage by inducing these gene expressions. In addition, lncRNAs are being explored for their role in fibrosis regulation and cardiac remodeling, which could offer new diagnostic strategies [54]. Additionally, the development of various molecular typing methods that target multiple copies or multigene families has enhanced the analysis of phylogenetic, taxonomic, and genetic studies [55]. 

## 6. ncRNAs as Emerging Biomarkers in the Diagnosis of Chagas Cardiomyopathy

Given that to date there is a lack of evidence on ncRNAs that can be reliably used for the diagnosis of Chagas disease, both acute and chronic, we discuss here the studies on Chagas cardiomyopathy that evaluate the sensitivity and specificity of ncRNAs.

During infection with *Trypanosoma cruzi*, changes occur in the expression of both coding and non-coding genes of the host cell, as well as in the expression of various ncRNAs, including miRNAs, which have been the most extensively studied. The types of miRNAs that undergo alterations depend on different factors such as the type of cell and the time of infection [57].

Research into circulating miRNAs has identified potential biomarkers for chronic Chagas disease and its associated cardiomyopathy. A study involving the sequencing of plasma samples from patients found higher expression of a group of miRNAs, including miR-Conting-1519, miR-Conting-3244, and miR-148a-3, in individuals with the disease [58]. Furthermore, a review by Gomes Ribeiro et al. compiled available information on the most expressed miRNAs during *T. cruzi* infection in patients with chagasic cardiomyopathy, identifying miR-21, miR-146b, miR-146a, and miR-145 as key molecules (Table 5). These specific miRNAs were linked to crucial pathological processes, including the immune response, fibrosis, and inflammation of heart tissues [59].

Recent studies on circulating miRNAs have provided strong evidence for their use as diagnostic tools in chronic Chagas cardiomyopathy (CCC). Two key studies, by Nonaka et al. (2019) [60] and Antonietti et al. (2023) [61], highlight the potential of specific miRNA biomarkers. Nonaka et al. (2019) identified miR-19a-3p as a standout diagnostic biomarker. It achieved an area under the ROC curve (AUC) of 0.77, with 67% sensitivity and 80% specificity for differentiating CCC patients from those with indeterminate forms of the disease [60]. The study also validated miR-29b-3p, showing moderate diagnostic performance (AUC = 0.70, 60% sensitivity, 70% specificity), and miR-21-5p, which had measurable but lower utility (AUC = 0.54, 57% sensitivity, 60% specificity). These miRNAs also correlated well with important clinical indicators. Positive correlations with cardiac dysfunction [miR-19a-3p: r = 0.47), NYHA functional class, and cardiac fibrosis (measured by cardiac MRI), and Inverse correlations with ejection fraction and left ventricular strain. In parallel, Antonietti et al. (2023) identified miR-130b-3p as a highly promising diagnostic biomarker, exhibiting superior discriminatory value (AUC = 0.79) [61]. They also found that miR-95-3p exhibited good diagnostic performance (AUC = 0.68). Both studies found that the identified miRNAs were consistently upregulated in CCC patients compared to healthy individuals, Chagas non-cardiomyopathy patients, and ischemic cardiomyopathy controls. This suggests their specificity for CCC diagnosis. Further bioinformatics analysis revealed that these miRNAs regulate genes involved in crucial cardiac processes underlying CCC, such as arrhythmia generation, cardiomegaly, and hypertrophy. Specifically, miR-95 targets CALM1 and miR-130b targets TSC1, both of which are associated with ventricular arrhythmias.

In addition, primary human cardiac fibroblasts have revealed that *T. cruzi* infection significantly alters the expression profile of piRNAs, suggesting their potential as biomarkers for Chagas disease. A study by Rayford et al. [62] identified 441 unique piRNAs that were differentially expressed during the early phase of parasitic infection. These researchers found that many of these piRNAs, such as hsa_piR_016828 and the novel npiR_17, target genes crucial in the fibrotic and inflammatory processes characteristic of Chagas disease, including ICAM1 and SMAD2. The up- or down-regulation of these piRNAs, e.g., npiR_167, that targets the pro-fibrotic transcription factor EGR1, suggested that these molecules could serve as markers in Chagas disease.

## 7. Limitations and Future Perspectives

**Figure 2:**
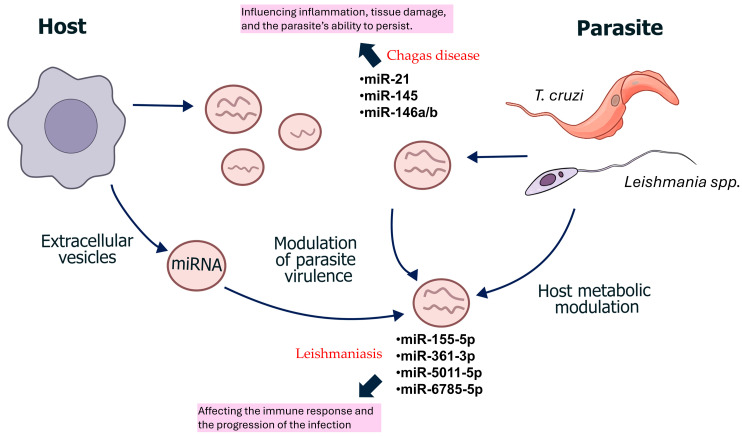
ncRNAs represent potential diagnostic biomarkers and regulatory molecules in host–parasite interactions in *Leishmania* and *Trypanosoma cruzi* infections.

Despite significant advances in understanding the regulatory roles of ncRNAs in *Leishmania* and *Trypanosoma cruzi* infections, their clinical translation as reliable biomarkers remains limited, Figure 2. Current studies are often exploratory, based on small sample sizes, heterogeneous experimental designs, and a lack of standardized methodologies for RNA extraction, normalization, and quantification. Furthermore, variations in host species, infection stages, and tissue origins complicate the reproducibility and comparability of results among studies.

A critical knowledge gap exists in differentiating ncRNAs linked to infection detection from those indicative of disease progression or organ-specific pathology [63]. In *T. cruzi*, most available data relate to cardiac alterations involvement during the chronic phase [64], while information on acute or asymptomatic infections remains scarce. Similarly, for *Leishmania,* the repertoire of ncRNAs (RNAome) varies between species and clinical forms, complicating their diagnostic application [50].

Future research should prioritize large-scale, multicenter studies using harmonized protocols and clinically relevant cohorts to validate non-coding RNA signatures. The combined use of transcriptomics, proteomics, and metabolomics could elucidate the mechanistic connections between ncRNAs derived from parasites and hosts. Finally, the development of cost-effective, point-of-care assays for ncRNA detection, particularly in endemic and resource-limited settings, represents a crucial step toward their implementation as practical biomarkers for neglected tropical diseases.

## 8. Conclusions

ncRNAs represent promising molecular biomarkers for both Leishmaniasis and Chagas disease. Current evidence highlights their potential to enhance diagnostic specificity and sensitivity, support disease staging, and predict therapeutic responses. Small RNA species derived from both parasites and host cells play critical roles in modulating pathogenesis, immune responses, and clinical outcomes. Several miRNAs—such as miR-155-5p and miR-361-3p in leishmaniasis, and miR-21, miR-145, and miR-146a/b in Chagas disease—have demonstrated promising diagnostic performance in clinical samples. However, the translation of these findings into routine clinical practice remains constrained by the lack of large-scale, standardized validation studies. Furthermore, lncRNAs represent an underexplored diagnostic resource that requires additional investigation to clarify their potential applications. Also, while small interfering RNAs are valuable research tools for understanding gene regulation and pathogenesis, they are not currently used as biomarkers or direct diagnostic tools for leishmaniasis or Chagas disease. In conclusion, integrating ncRNA biomarker profiling into current diagnostic workflows could revolutionize the clinical management of these neglected diseases, facilitating earlier diagnosis, more accurate prognostic assessment, and targeted therapeutic monitoring. Future research should focus on large, multicenter studies to validate ncRNA signatures, assess cost-effectiveness, and establish standardized methodologies suitable for implementation in endemic regions.

## Figures and Tables

**Table 1:** Diagnostic Comparison of Cutaneous, Mucocutaneous, and Visceral Leishmaniasis [12,13].

Characteristic	Cutaneous Leishmaniasis	Mucocutaneous Leishmaniasis	Visceral Leishmaniasis
**Causative agents**	*L. major*, *L. tropica* (Old World); *L. mexicana*, *L. amazonensis*, *L. guyanensis*, *L. panamensis*, *L. braziliensis* (Americas)	Mainly *L. braziliensis*	*L. donovani*, *L. infantum* (Old World); *L. infantum*/*L. chagasi* (Americas); occasional cases caused by viscerotropic strains of *L. tropica*
**Endemic regions**	Old World: Africa, Middle East, Asia; New World: Central and South America	Primarily South America: Brazil, Peru, Bolivia; also seen in Colombia, Ecuador, Paraguay, and Venezuela	Old World: India, Pakistan, China, Africa, Mediterranean region; New World: mainly Brazil
**Pathogenesis**	Localized skin infection with an inflammatory reaction at the inoculation site	Spread or metastasis of the parasite from cutaneous lesions to nasopharyngeal and/or oropharyngeal mucosal tissues	Systemic dissemination to organs of the reticuloendothelial system (liver, spleen, bone marrow)
**Clinical manifestations**	Single or multiple; typically on exposed areas (face, arms, legs)	Progressive destruction of mucous membranes and soft tissues: nose, mouth, pharynx, eyelids, can cause severe disfigurement and respiratory/nutritional difficulties	Prolonged fever, weight loss, hepatosplenomegaly, anemia, pancytopenia, immunosuppression; HIV coinfection increases severity
**Clinical course**	Usually self-limiting, but may progress to mucocutaneous disease (especially with *L. panamensis* and *L. braziliensis*)	Chronic evolution can develop months or years after initial cutaneous lesions; high risk of complications	Potentially fatal if untreated; immunosuppression increases the risk of opportunistic infections.
**Diagnosis**	Visualization of amastigotes (Microscopic evaluation), culture, PCR (conventional and real-time); biopsy in diffuse cases	Parasitological and molecular diagnosis (DNA sequencing analysis; also cellulose acetate electrophoresis); evaluation of mucosal involvement	Combination of clinical findings, serology (ELISA, DAT), parasite visualization (smear, bone marrow aspirate), PCR (conventional and real-time)
**Treatment**	Local therapy in mild cases; systemic therapy in extensive disease or mucocutaneous risk	Prolonged systemic treatment; limited response, risk of recurrence	Requires immediate systemic therapy; management is more complex in HIV-coinfected patients
**Prognosis**	Good in most cases, especially with early treatment	Guarded; high morbidity and disfigurement; mortality mainly due to secondary infections and malnutrition	Severe if untreated; with appropriate therapy, mortality decreases significantly

**Abbreviations:** PCR, polymerase chain reaction; ELISA, enzyme-linked immunosorbent assay; DAT, direct agglutination test.

**Table 2:** Diagnostic Methods for Chagas Disease [14,15].

Diagnostic Aspect	Acute Phase	Chronic Phase *
**Morphology**	Detection of trypomastigotes in circulating blood or CSF via direct microscopy. Thick and thin blood smears stained with Giemsa or H&E are used. Amastigotes may also be identified in biopsy specimens.	Trypomastigotes rarely found in blood. Serologic tests are preferred for diagnosis. Amastigotes can occasionally be identified in tissue biopsies.
**Serology** (*Antibody Detection*)	There are no accurate IgM based methods available for serological diagnosis at the acute phase.	Primary diagnostic method due to low parasitemia. Requires ≥ 2 serologic tests targeting different antigens. CDC protocols: - First line: FDA-cleared enzyme immunoassay (EIA) + TESA immunoblot. - If discordant: request second sample and repeat testing. - If still discordant: use immunofluorescence assay (IFA) as a “tie-breaker.” Sensitivity and specificity vary across commercially available tests.
**Molecular Testing**	Recommended in suspected acute infections, congenital cases, transfusion/transplant transmission, or laboratory exposures. CDC method: real-time PCR using two assays (TCZ and MNC). Acceptable specimens: EDTA blood (≥2.2 mL), heart biopsy tissue, and CSF if CNS involvement is suspected.	Generally not used routinely for diagnosis but may be performed in reactivation cases linked to immunosuppression (e.g., HIV). Serology remains the preferred method.

**Abbreviations:** CSF, Cerebrospinal fluid; H&E, Hematoxylin and eosin stain; EIA, Enzyme immunoassay; TESA, Trypomastigote excreted-secreted antigen immunoblot; IFA, Indirect immunofluorescence assay; PCR, Polymerase chain reaction; TCZ, *T. cruzi*–PCR target; MNC, Minicircle DNA PCR target; EDTA, Ethylenediaminetetraacetic acid. * The WHO advises using at least two assays with different principles to confirm a positive serological result for *T. cruzi* infection [16].

**Table 3:** ncRNAs for the diagnosis of Leishmaniasis and Chagas disease [42,43,46,47,48].

Feature	tRNA-Derived Small RNAs in *T. cruzi*	tRNA-Derived Small RNAs in Leishmania	lncRNAs
Size	31–40 nt	20–40 nt	>200 nt
Precursor	Mature tRNAs cleaved at anticodon loop (tRNA halves) or at D loop, T loop, anticodon loop, or 3′ leader (tRFs)	Mature tRNAs	Primary transcripts from RNA Pol I, II, or III
Processing enzymes	Dicer is absent in *T. cruzi*	Most Leishmania lack RNAi machinery	Standard RNA processing machinery
Associated proteins	TcPIWI-tryp (trypanosomatid-specific PIWI-like protein)	AGO/PIWI homolog (present in RNAi-deficient species)	Spliceosome, transcriptional regulators, RNA stability proteins
RNAi machinery	Absent (no canonical Dicer or Argonaute)	Absent in most species; retained only in *L. braziliensis* (Viannia subgenus)	N/A
Functional notes	TcPIWI-tryp binds small RNAs derived from structural RNAs; *T. cruzi* lost RNAi during evolution.	*L. major*, *L. donovani*, *L. infantum* lack RNAi; miRNA-like elements computationally predicted but not validated.	Differentially expressed during infection; potential regulatory roles in host–pathogen interactions.

**Table 4:** Potential ncRNA in the diagnosis of Leishmaniasis.

ncRNA Type	Biomarker(s)	Sample/Clinical Context	Sensitivity	Specificity	AUC
miRNA	miR-155-5p, miR-5011-5p, miR-6785-5p, miR-4795-3p [55]	Lesion biopsies (*L. major*, *L. tropica*)	86–100%	100%	0.92–1.00
miRNA	miR-361-3p [54]	Lesion biopsies (*L. braziliensis*)	81.20%	100%	Not reported
lncRNA	MALAT1, NUTM2A-AS1, LINC00963, others [56]	Serum (visceral or cutaneous leishmaniasis patients)	Not quantified	Not quantified	Not quantified

**Table 5:** Circulating microRNAs as emerging biomarkers in Chagas Cardiomyopathy.

ncRNA Type	Biomarker	Sample/Clinical Context	Sensitivity	Specificity	AUC (ROC)
miRNA [60]	miR-19a-3p	Serum	67%	80%	0.77
	miR-21-5p		57%	60%	0.54
	miR-29b-3p		60%	70%	0.7
	miR-199b-5p		67%	57%	0.57

## Data Availability

No new data were created or analyzed in this study.

## Abbreviations

The following abbreviations are used in this manuscript:

*T. cruzi*	*Trypanosoma cruzi*
miRNAs	microRNAs
ncRNAs	non-coding RNAs
lncRNAs	long non-coding RNAs
PAHO	Pan American Health Organization
piRNAs	PIWI-interacting RNAs
RNAome	repertoire of ncRNAs
SANRA	Scale for the Assessment of Narrative Review Articles
siRNAs	short interfering RNAs
VL	visceral leishmaniasis
WHO	World Health Organization
